# Pre-ductal and Post-ductal Oxygen Saturation Trends in Neonates: A Cross-Sectional Observational Study

**DOI:** 10.7759/cureus.96906

**Published:** 2025-11-15

**Authors:** Ganesh S Mena, Vikram Sakaleshpur Kumar, Amulya V, Samadhara V Jogi, Nimishashree K Srinivas

**Affiliations:** 1 Pediatrics and Child Health, Subbaiah Institute of Medical Sciences, Shivamogga, IND; 2 Pediatric Medicine, Sarji Mother and Child Care Hospital, Shivamogga, IND; 3 Pediatrics, Subbaiah Institute of Medical Sciences, Shivamogga, IND

**Keywords:** maternal comorbidities, neonatal oxygenation, newborn adaptation, post-ductal spo₂, pre-ductal spo₂, pulse oximetry

## Abstract

Background

The transition from fetal to neonatal life involves significant physiological changes, particularly in arterial oxygen saturation (SpO₂), which can be visually observed as skin color changes. However, visual assessment alone is prone to variability. Pulse oximetry offers a reliable, non-invasive method to monitor SpO₂ levels, providing critical information on newborn oxygenation and aiding in the early detection of critical congenital heart diseases (CCHD).

Methods

This observational cross-sectional study was conducted at a tertiary care center over 18 months, involving 250 newborns. Pre-ductal and post-ductal SpO₂ were measured using pulse oximeters at 2, 3, 4, 5, 10, and 15 minutes or until levels surpassed 90%. Data analysis was performed using Student's t-test and the Mann-Whitney U test, with a significance threshold of p < 0.05.

Results

The mean time for pre-ductal SpO₂ to reach 90% was 8.68 minutes, while post-ductal SpO₂ took 9.05 minutes. The time for pre-ductal and post-ductal SpO₂ to equalize was 11.57 minutes. Maternal age, gestational age, birth weight, and maternal hemoglobin levels had minimal impact on SpO₂ stabilization.

Conclusion

This study highlights the critical role of oxygen supplementation in accelerating SpO₂ stabilization and the influence of maternal comorbidities on neonatal oxygenation. Despite minimal impact from other factors, targeted monitoring and intervention are essential, particularly for newborns born to mothers with health conditions. These insights emphasize the need for tailored neonatal care strategies to optimize outcomes during this critical period of adaptation. Targeted oxygen supplementation and maternal comorbidity screening may help optimize neonatal transition.

## Introduction

The transition from fetal to neonatal life is a critical period marked by complex physiological adaptations, particularly within the circulatory and respiratory systems. One of the most significant changes includes the closure of fetal circulatory pathways, such as the ductus arteriosus, and the establishment of pulmonary circulation suitable for postnatal life [1,2]. As the lungs aerate and pulmonary vascular resistance drops, blood flow patterns shift accordingly, resulting in rapidly changing oxygen saturation (SpO₂) levels [3,4].

Pulse oximetry is highlighted as a key tool in this study. Although arterial blood gas (ABG) analysis remains the gold standard for assessing SpO₂, pulse oximetry serves as a reliable alternative for continuous monitoring in clinical and neonatal settings. It is a non-invasive, cost-effective, and reliable method for continuously monitoring SpO₂ levels in newborns. Its ease of use, minimal discomfort to the baby, and high detection rates for duct-dependent circulation make it an ideal screening tool in neonatal care. During the transition from fetus to newborn, arterial SpO₂ undergoes dramatic changes, which can be clinically observed as changes in skin colour. However, this visual assessment is prone to intra- and inter-observer variability, reinforcing the need for objective tools such as the pulse oximeter [5,6].

SpO₂ is approximately 40% in utero and rises rapidly after birth, typically exceeding 90% within the first few minutes of life. Pre-ductal oxygen saturation, measured at the right hand or wrist, reflects blood oxygenation before it passes through the ductus arteriosus. Post-ductal saturation, measured at the foot or opposite hand, reflects oxygenation after blood has passed through the ductus [7]. Initially, pre-ductal saturation tends to be higher due to systemic-to-pulmonary shunting, but as the ductus closes and pulmonary circulation improves, the two values typically converge [7,8]. Persistent differences between them may indicate underlying cardiac or pulmonary pathology requiring immediate attention [1-8].

Simultaneous monitoring of pre- and post-ductal saturation is especially vital in detecting conditions such as persistent pulmonary hypertension of the newborn (PPHN) and critical congenital heart disease (CCHD), some of which remain asymptomatic in the early neonatal period and may be missed by physical examination alone. Indeed, studies show that up to 30-50% of CCHD cases can go undetected without pulse oximetry [6-13]. Hence, pulse oximetry is not only vital for oxygen titration during resuscitation, as recommended by the American Academy of Pediatrics (AAP) and the American Heart Association (AHA), but also as a primary screening tool for life-threatening anomalies [14]. SpO₂ should be targeted within the range of 91-95% when receiving oxygen therapy, in both preterm and term neonates [15].

Preterm newborns often have different physiological needs compared with term newborns and are more susceptible to oxygen toxicity, which can have harmful effects on developing tissues [16,17]. However, there is a lack of specific SpO₂ ranges tailored for preterm infants in many guidelines, including those provided by the International Liaison Committee on Resuscitation (ILCOR), established in 1992 to develop global consensus on evidence-based emergency cardiovascular care and resuscitation practices, with representation from the AHA, AAP, and other international councils. Studies, such as a prospective observational study from Northern India, show that preterm neonates tend to have slower rises in SpO₂ compared with term neonates, underscoring the need for differentiated management strategies [17,18]. Moreover, differences in SpO₂ levels based on gestational age, delivery method (vaginal vs. caesarean), and regional variations highlight the need for India-specific reference ranges and protocols [19]. Many current guidelines are based on Western populations, which may not reflect the diverse ethnic, geographic, and socioeconomic landscape in India. Despite its proven utility, studies on pre- and post-ductal SpO₂ trends in Indian newborns remain scarce. Our study was specifically chosen and designed to address this critical gap. It explores a simple, effective, and underutilised method of CCHD detection by evaluating pre- and post-ductal SpO₂ trends during the neonatal transition phase in Indian newborns. By generating context-specific data, we aim to inform future clinical practices and contribute to improved neonatal outcomes in similar settings.

## Materials and methods

This observational cross-sectional study was conducted from August 2022 to February 2024 in a tertiary care center, after obtaining ethical clearance from the Institutional Ethics Committee. Informed written consent was obtained from the parents of all enrolled neonates. All live-born singleton neonates delivered at the hospital during the study period were screened. Exclusion criteria included twin pregnancies and neonates with a family history of hemoglobinopathies, to avoid confounding SpO₂ profiles.

A total of 250 neonates met the inclusion criteria and were enrolled in the study. The sample size was estimated using a power analysis formula to detect a statistically significant difference in SpO₂ trends between pre- and post-ductal measurements. Based on a 95% confidence level (Zα/2 = 1.96), 80% power (Zβ = 0.84), an estimated variance (σ²) from previous literature, and a clinically relevant difference (Δ) in SpO₂ levels, the required sample size was calculated. To strengthen statistical validity and allow for exclusions or missing data, all eligible neonates delivered during the study period were included.

Immediately after cord clamping, trained personnel placed pulse oximeter probes (MINDRAY MEC 2000) on the neonate’s right hand (pre-ductal) and foot (post-ductal). Plethysmographic waveform confirmation was used to ensure reliable signal detection. SpO₂ readings were recorded at 2, 3, 4, 5, 10, and 15 minutes post-birth, or until pre-ductal saturation exceeded 90%. Two separate observers handled clinical care and data collection to minimize interference and ensure objectivity. Standard neonatal care, as per WHO guidelines, was maintained throughout without any compromise.

The procedure was subject to certain limitations. Motion artefacts, local hypoxemia, hypothermia, and poor peripheral perfusion are known factors that can interfere with pulse oximeter accuracy. Every effort was made to reduce these, including the use of well-trained staff and ensuring optimal probe placement with verified waveforms. However, some degree of measurement bias remains possible. Observer bias may have occurred, as complete blinding was not feasible. Additionally, being a single-center study, the findings may not be generalizable to other populations. Selection bias was also possible, as only inborn neonates were included, excluding outborn or high-risk deliveries.

Statistical analysis was performed using SPSS version 23.0. Parametric data were analyzed using Student’s t-test, and non-parametric data with the Mann-Whitney U test. Pearson’s correlation coefficient was used to assess the relationship between pre- and post-ductal saturation levels. A p-value <0.05 was considered statistically significant. Confounding variables such as maternal hemoglobin levels, gestational age, birth weight, and comorbidities were recorded and examined for their influence on SpO₂ trends. Missing data were negligible, and no imputation was necessary.

## Results

**Figure 1:**
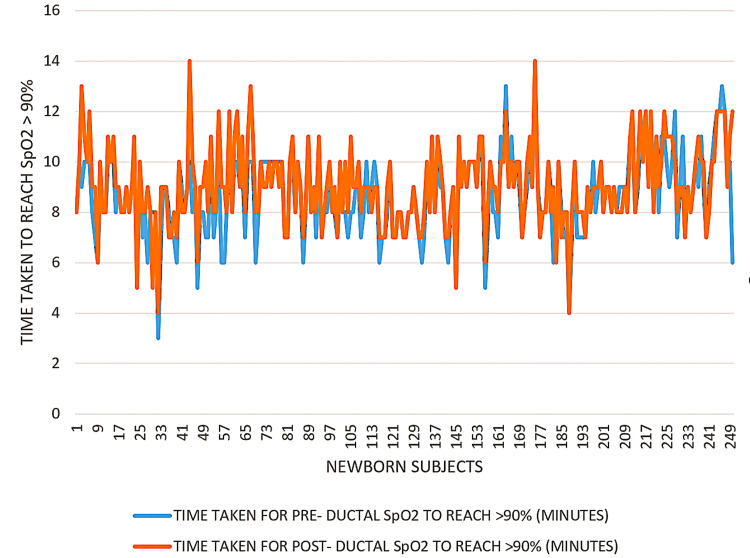
Line diagram showing time taken for pre-ductal and post-ductal SpO₂ to reach >90%. This figure illustrates the comparative progression of oxygen saturation levels over time at pre-ductal and post-ductal sites in neonates. Data points represent mean ± SD values at the specified time intervals. A p-value < 0.05 was considered statistically significant. SpO₂: Oxygen saturation.

A total of 250 newborns were included in the study. The primary objective was to determine the time taken for pre-ductal and post-ductal SpO₂ to reach ≥90% and the time required for both values to equalise. The mean time for pre-ductal saturation to cross the 90% threshold was 8.68 ± 1.59 minutes, while post-ductal saturation took slightly longer, averaging 9.05 ± 1.63 minutes. The mean time for equalisation of pre- and post-ductal values was 11.57 ± 1.78 minutes, with a median of 12 minutes (IQR 3) and a range of 6 to 15 minutes. This progression pattern, including the correlation between the two readings (r = 0.797, p < 0.001), is detailed in Table 1 and illustrated in Figure 1.

**Table 1:** Time to reach ≥90% SpO₂ and equalization. Data are presented as mean ± SD, median (IQR, a measure of statistical dispersion), and range. Statistical significance was assessed using Student’s t-test, with p < 0.05 considered significant. SpO₂: Oxygen saturation.

Variable	Mean ± SD	p-value	Additional statistics
Pre-ductal SpO₂ (min)	8.68 ± 1.588	<0.001	-
Post-ductal SpO₂ (min)	9.05 ± 1.633	-	-
Equalization time (min)	11.57 ± 1.78	<0.001	Median (IQR): 12 (3); Range: 6-15; 95% CI: 11.34-11.80

The influence of maternal age on SpO₂ trends was assessed by grouping mothers into ≤20 years, 21-30 years, and 31-40 years. Although there appeared to be a slightly quicker rise in saturation in neonates of younger mothers, the differences were not statistically significant (p = 0.209 pre-ductal; p = 0.845 post-ductal). Similarly, parity showed no substantial effect, with comparable timings observed in neonates of primigravida and multigravida mothers (Table 2). Gestational age was analysed, and while preterm neonates reached saturation slightly earlier than term neonates, the difference did not reach statistical significance. Birth weight also did not significantly impact the time to achieve target saturation levels. Across birth weight groups (≤2500 g, 2600-3500 g, ≥3600 g), the variation in mean SpO₂ stabilisation times was minimal, and statistical comparisons confirmed the lack of a meaningful association (p > 0.05 for both pre- and post-ductal times).

**Table 2:** Maternal and neonatal factors vs. time to reach SpO₂ ≥90%. Data are presented as frequency (n), mean ± SD, and corresponding p-values (from Student’s t-test and Mann-Whitney U test), with p < 0.05 considered significant. LSCS: Lower Segment Caesarean Section; SGA: Small for Gestational Age; AGA: Appropriate for Gestational Age; LGA: Large for Gestational Age; SpO₂: Oxygen saturation; Hb: Hemoglobin.

Factor	Group	Frequency (n)	Pre-ductal (Mean ± SD)	Post-ductal (Mean ± SD)
Maternal age (years)	<20	5	7.20 ± 1.304	8.60 ± 1.140
	21-30	213	8.68 ± 1.597	9.03 ± 1.685
	31-40	32	8.97 ± 1.470	9.28 ± 1.326
p-value			0.209	0.845
Parity	Primigravida	122	8.57 ± 1.705	8.94 ± 1.731
	Multigravida	128	8.79 ± 1.467	9.16 ± 1.534
p-value			0.241	0.7
Gestational age	Early preterm	2	8.09 ± 1.411	8.59 ± 1.403
	Late preterm	22	10.50 ± 0.707	11.00 ± 1.414
	Term	226	8.73 ± 1.593	9.08 ± 1.645
p-value			0.125	0.381
Maternal Hb (g/dL)	8-10.9	71	8.87 ± 1.673	9.31 ± 1.729
	11-11.9	97	8.65 ± 1.665	8.93 ± 1.550
	>12	82	8.56 ± 1.415	8.98 ± 1.640
p-value			0.097	0.077
Neonatal gender	Male	119	8.64 ± 1.721	9.00 ± 1.780
	Female	131	8.73 ± 1.463	9.10 ± 1.493
p-value			0.963	0.967
Appropriateness for gestational age	SGA	50	8.50 ± 0.919	9.02 ± 1.911
	LGA	2	8.00 ± 1.414	10.00 ± 0.000
	AGA	198	8.74 ± 1.502	9.06 ± 1.569
p-value			0.424	0.158
Maternal comorbidities	Yes	30	8.10 ± 1.845	8.76 ± 1.538
	No	220	8.77 ± 2.046	9.09 ± 1.571
p-value			0.047	0.379
Oxygen supplementation given	Yes	97	7.88 ± 1.589	8.26 ± 1.609
	No	153	9.20 ± 1.367	9.57 ± 1.441
p-value			<0.001	<0.001
Mode of delivery	Spontaneous vaginal	70	8.83 ± 1.382	9.14 ± 1.427
	Induced vaginal	10	8.55 ± 1.440	9.27 ± 1.104
	LSCS	170	9.10 ± 1.500	9.30 ± 1.500
p-value			0.872	0.902

Maternal hemoglobin levels were considered a potential influencing factor, but no significant relationship was found. Neonates of mothers with hemoglobin <11 g/dL showed slightly longer times to reach saturation; however, these trends did not attain statistical significance (p = 0.097 pre-ductal; p = 0.077 post-ductal). When neonatal sex was assessed, no significant differences were noted between male and female neonates with respect to SpO₂ dynamics.

Mode of delivery was another parameter of interest. Although not statistically significant, neonates born via caesarean section showed a trend toward delayed oxygenation compared with those delivered vaginally, with mean pre-ductal times of 9.05 vs. 8.41 minutes, respectively (p = 0.079). Among vaginal deliveries, no significant differences were observed between spontaneous and induced labour groups (Table 2).

A notable finding was the influence of maternal comorbidities on neonatal SpO₂ stabilisation. Newborns of mothers with conditions such as gestational diabetes mellitus (GDM) and hypothyroidism showed delayed pre-ductal saturation compared with those without comorbidities (8.10 vs. 8.77 minutes, p = 0.047). Post-ductal saturation differences were not significant (p = 0.379). Furthermore, the impact of oxygen supplementation was clearly significant. Neonates who received supplemental oxygen reached pre-ductal saturation ≥90% in 7.88 minutes compared with 9.20 minutes for those who did not (p < 0.001). A similar effect was seen for post-ductal saturation, achieved in 8.26 minutes with oxygen supplementation versus 9.57 minutes without (p < 0.001). Oxygen supplementation was therefore a major determinant, significantly improving the time to reach target saturation levels. This supports existing recommendations for early oxygen support when warranted and should inform neonatal resuscitation protocols in similar settings.

Lastly, descriptive statistics for participant demographics and clinical characteristics, including maternal age distribution, parity, gestational age, neonatal sex, and the prevalence of maternal comorbidities, are summarised in Table 2. A breakdown of specific maternal comorbidities, such as GDM (4.4%), hypothyroidism (4%), and pre-eclampsia (1.6%), is presented in Table 3. GDM refers to high blood glucose developing during pregnancy due to insulin resistance; although it may resolve after delivery, it increases the future risk of type 2 diabetes for both mother and child. Overall, the results reinforce the physiological variability during the neonatal transition and highlight oxygen supplementation and maternal comorbidities as the most relevant influencing factors in early postnatal SpO₂ trends.

**Table 3:** Types of maternal comorbidities. Data are presented as frequency (n) and percentage (%). GDM: Gestational diabetes mellitus; PIH: Pregnancy-induced hypertension.

Maternal comorbidities	Frequency (n)	Percentage (%)
Chronic hypertension and pre-eclampsia	1	0.40%
GDM	11	4.40%
GDM with pregnancy-induced hypertension (PIH)	1	0.40%
Hypothyroidism	10	4.00%
Hypothyroidism with GDM	1	0.40%
PIH	3	1.20%
PIH with pre-eclampsia	1	0.40%
Pre-eclampsia	1	0.40%
None	221	88.00%
Total	250	100.00%

## Discussion

This study evaluated the temporal trends in pre-ductal and post-ductal SpO₂ in newborns during the critical transition period following birth. We observed that pre-ductal SpO₂ levels reached >90% at an average of 8.68 minutes, whereas post-ductal saturation required approximately 9.05 minutes, with equalisation occurring around 11.57 minutes. These findings align with previous reports emphasising the gradual rise in neonatal SpO₂ as the pulmonary circulation adapts to extrauterine life.

Our results are consistent with those of Tiwari S et al., who reported a mean equalisation time of 11.46 minutes among term neonates, with minimal influence from factors such as gestational age, birth weight, and parity [20]. Similarly, Odudu LA et al. demonstrated comparable SpO₂ levels in term and late preterm low-birth-weight neonates, suggesting that minor physiological differences may not significantly impact transitional SpO₂ trends [21]. These parallels support the notion that neonatal circulatory adaptation is a robust process largely independent of certain maternal or fetal variables in uncomplicated cases.

We noted that maternal age, parity, gestational age, and birth weight did not significantly affect SpO₂ trends in our cohort. However, maternal comorbidities such as hypothyroidism and gestational diabetes were associated with subtle delays in SpO₂ stabilisation. While these associations are not widely explored in existing studies, they merit further investigation, particularly given the increasing prevalence of such conditions in obstetric populations. As this was a single-centre study, the findings may not fully represent variations across different demographic or regional groups. Future multi-centre studies with larger and more diverse samples are recommended to validate these observations.

The mode of delivery was another factor influencing oxygenation patterns. Although statistical significance was not observed, caesarean-delivered neonates tended to have a slightly delayed rise in SpO₂ compared with those delivered vaginally. This is consistent with the findings of Mariani G et al., who reported that SpO₂ often remains below 90% for the first five minutes, particularly following caesarean section [22]. The lack of thoracic compression during operative delivery and altered hormonal responses may explain this difference.

An interesting observation in our study was the occasional occurrence of higher post-ductal than pre-ductal saturations during early measurements. This pattern, although counterintuitive, is physiologically plausible and has been previously reported by Jegatheesan P et al., who documented variable SpO₂ patterns in healthy neonates during transitional circulation [23]. Such findings underscore the dynamic nature of neonatal oxygenation and highlight the importance of simultaneous monitoring at both pre- and post-ductal sites.

Our study reinforces the value of pulse oximetry as a vital, non-invasive tool for monitoring SpO₂ trends in neonates. Beyond basic monitoring, its role in screening for critical congenital heart diseases (CCHDs) is increasingly recognised. As demonstrated by Rüegger C et al., pulse oximetry enables early identification of duct-dependent cardiac lesions, even in asymptomatic newborns [24]. Early detection of such anomalies is crucial, particularly in settings where clinical examination alone may fail to identify subtle signs.

In addition, Reddy SC et al. recently reported similar SpO₂ patterns, further supporting the reproducibility of our observations across different Indian populations [25]. The trend of delayed SpO₂ rise in caesarean-delivered newborns observed in our study is consistent with findings by Zanardo V et al., who noted that elective caesarean section adversely affects initial SpO₂ due to delayed clearance of fetal lung fluid and altered hormonal physiology [26].

Beyond its monitoring role, pulse oximetry has been well established as a screening tool. Our findings highlight its importance in detecting subtle early physiological differences in newborns who appear clinically normal. Gaonkar PM et al. underscored its pivotal utility in identifying critical congenital heart diseases in asymptomatic neonates, especially in rural settings where relying solely on physical examination may be inadequate.

In conclusion, our study adds to the expanding body of evidence supporting pulse oximetry as an essential component of routine neonatal assessment. It also emphasises the need for population-specific data to guide the interpretation of SpO₂ values, particularly in diverse regions such as India, where ethnic, environmental, and healthcare-system differences may influence neonatal outcomes.

## Conclusions

This study on the trends of pre-ductal and post-ductal SpO₂ in newborns provides valuable insights into the neonatal transition to extrauterine life. Our findings revealed that pre-ductal SpO₂ levels generally stabilise slightly earlier than post-ductal levels, with both measures equalising within approximately 12 minutes. Importantly, factors such as maternal age, parity, gestational age, neonatal sex, and birth weight had minimal impact on the time taken to reach 90% SpO₂, underscoring the robustness of neonatal oxygenation processes across diverse demographic and clinical profiles. However, the study also highlights areas requiring particular attention in neonatal care. Maternal comorbidities were associated with a statistically significant delay in the time taken for pre-ductal SpO₂ to reach 90%, suggesting that newborns of mothers with conditions such as hypertension, diabetes, or thyroid disease may require closer monitoring and potentially earlier intervention to ensure optimal oxygenation. Additionally, the study emphasizes the beneficial impact of oxygen supplementation, which significantly reduced the time required to reach 90% SpO₂, reinforcing its role as a critical intervention in neonatal resuscitation and care.

Overall, these findings contribute to a better understanding of neonatal oxygenation patterns and emphasize the need for tailored strategies in managing newborns, particularly those at higher risk due to maternal health conditions. By ensuring timely and effective monitoring and intervention, healthcare providers can improve the outcomes for newborns during this critical period of physiological adaptation
